# Notes and Comments

**Published:** 1898-05

**Authors:** 


### Notes and Comments.[1]

NEURECTOMY FOR TIC DOULOUREUX.—In a paper before the Mississippi Valley Medical Society, Professor Bernays reports the following case where neurectomy was performed for the relief of a severe case of tic douloureux, and refers to the value of antikamnia as an obtundent. He says, in part, "The patient has one sister who suffered from emotional insanity; otherwise the family history is good. Previous health excellent. The present trouble began with a severe neuralgic toothache, localized in the right lower molars. Paroxysms of pain were of daily occurrence, and most severe in the mornings about breakfast time. The pain subsided temporarily whenever the teeth were pressed firmly together or upon any substance held between them, but only to return when the pressure was withdrawn. The presence of anything cold in the mouth immediately produced the most exquisite pain; moderate heat produced a soothing effect. After two months the pain became continuous, and five teeth were extracted at different times without in any way relieving the trouble. On the contrary, the pain increased in severity until October, when it ceased entirely for a period of two weeks. A recurrence then took place, together with an involvement of the parts supplied by the second branch of the fifth nerve. She had strenuously avoided the use of narcotics, but during the more active periods of pain, antikamnia in ten-grain doses was found to be an efficacious obtunder." After describing the neurectomy, Professor Bernays says, "Eight weeks have now elapsed since the operation, and no recurrence of the trouble has taken place."

---

COCAINIZING PULPS FOR REMOVAL.—The removal of dental pulps, which is never an undreaded operation by the patient, and not always an easy one for the operator, can often be accomplished very satisfactorily to both. Dr. J. Y. Crawford, of Nashville, says, "Of all the operations in minor surgery demanding local anæsthesia, the removal of a live pulp most demands it. Having

---

[1] The assistant editor solicits contributions for this department,—new methods, new remedies and formulas, or any short practical note which may prove of value to the practitioner or student. Address 1338 Walnut Street, Philadelphia.

decided through diagnosis by exclusion that it is clearly a case of pulpitis and that the pulp must be removed, put on the rubber dam, dry the cavity, wipe it out with bichloride of mercury 1 to 3000, dry again, and repeat several times. When satisfied that it is sterilized, peel away the dentine carefully until you get a tiny exposure, then take a little muriate of cocaine, ten-per-cent. solution, on a tiny wad of cotton, and lay it over the point of exposure. With your fingers form of wax a lid for the cavity of the tooth, fit it over and convert the tooth into a force-pump, forcing in the cocaine. Then trim away more dentine, enlarge the exposure, and work your pump again, forcing in more cocaine; you will find that you can remove the pulp painlessly and pull it clear out of the lingual canal, which will offer no resistance. Now, before going any further, cork up the canal to prevent any *débris* entering. Now work in more cocaine, and with a broach remove the pulp from the next canal and fill that up, and so on. That tooth is worth all the pains you can take with it, and you want to keep the canals in an aseptic condition, preventing the entrance of any *débris* from the dentine you will remove in preparing the cavity for filling. That pumping process will enable you to anæsthetize the pulp so that you can remove it without pain to the patient, and you will not want any more arsenic. In case of hemorrhage, the application of the bichloride of mercury solution will arrest it at once."—*American Dental Weekly.*

---

A Good Suggestion.—We are of the opinion that the more the dentist knows of surgery and medicine the higher will be his appreciation of the surgeon and physician, and the more careful and the more able will he be to conduct his dental practice on the lines of his specialty, and avoid the pitfalls of a "little knowledge." —*Journal of the British Dental Association.*

---

Embarrassing Effects of Cocaine.—The *Dominion Dental Journal* reports a case where a patient after having a tooth extracted was arrested on account of the influence of the cocaine used as a local anæsthetic. When called upon to extract the tooth the dentist injected a few drops of cocaine into the gums. The operation went on successfully, but hardly had the patient left the office of the practitioner when a nervous excitation seized upon him, and without any reason led him to the palace of the governor, with the intention, as he shouted out, "to die in his arms." With great difficulty he was taken to the police head-quarters and kept for a few hours until his mind was in a normal state, when he was dismissed after having given an explanation.

---

Is the Use of Tobacco Injurious to the Teeth?—In an article on "Nicotiana" in the *Dominion Dental Journal*, Dr. A. C. Cogswell says, "The premature loss of teeth to those who use tobacco in any form, and more especially to excess, by many can be proved beyond a shadow of doubt. To those of the profession who are called to operate on the mouths and teeth of such persons, it is needless to say it is often found that the odor from such mouths is steeped with the last fumes of the pipe, the teeth blacker than the ace of spades, especially on the lingual surface, covered with nicotine, and gums spongy and receding from the necks of the teeth, usually attacking first the upper molars, causing exposure of the palatal roots, loosening of the same, and attended frequently with general elongation, pyorrhœa, and premature loss." While the writer does not use tobacco in any form, he has examined the mouths of many who were addicted to it, and has yet to see these deleterious effects. In fact, the conditions have always been quite the opposite.

